# Development of computer adaptive testing for measuring depression in patients with cancer

**DOI:** 10.1038/s41598-022-12318-x

**Published:** 2022-05-17

**Authors:** Ken Kurisu, Masayuki Hashimoto, Tetsuro Ishizawa, Osamu Shibayama, Shuji Inada, Daisuke Fujisawa, Hironobu Inoguchi, Haruki Shimoda, Shinichiro Inoue, Asao Ogawa, Tatsuo Akechi, Ken Shimizu, Yosuke Uchitomi, Yutaka Matsuyama, Kazuhiro Yoshiuchi

**Affiliations:** 1 Department of Stress Sciences and Psychosomatic Medicine, Graduate School of Medicine, The University of Tokyo, 7-3-1, Hongo, Bunkyo-ku, Tokyo, 113-8655 Japan; 2 Department of Psychosomatic Medicine, Sunagawa City Medical Center, Sunagawa, Hokkaido Japan; 3 Department of Psychosomatic Medicine, Yokohama Rosai Hospital, Yokohama, Kanagawa Japan; 4 Department of Psychosomatic Medicine, Kindai University Hospital, Osakasayama, Osaka Japan; 5 Department of Neuropsychiatry and Palliative Care Center, Keio University School of Medicine, Tokyo, Japan; 6 Department of Psycho-Oncology, National Cancer Center Hospital East, Kashiwa, Chiba Japan; 7 Department of Psycho-Oncology, National Cancer Center, Tokyo, Japan; 8 Department of Hygiene and Preventive Medicine, School of Medicine, Iwate Medical University, Iwate, Japan; 9 Department of Mental Health, The University of Tokyo, Tokyo, Japan; 10 Department of Neuropsychiatry, Okayama University, Okayama, Japan; 11 Center for Psycho-Oncology and Palliative Care, Nagoya City University Hospital, Nagoya, Aichi Japan; 12 Department of Psychiatry and Cognitive-Behavioral Medicine, Nagoya City University, Graduate School of Medical Sciences, Nagoya, Aichi Japan; 13 Department of Psycho-Oncology, Cancer Institute Hospital, Tokyo, Japan; 14 Innovation Center for Supportive, Palliative and Psychosocial Care, National Cancer Center, Tokyo, Japan; 15 Department of Biostatistics, School of Public Health, Graduate School of Medicine, The University of Tokyo, Tokyo, Japan

**Keywords:** Quality of life, Depression

## Abstract

The usefulness of depression scales for patients with cancer based on item response theory (IRT) and computer adaptive testing (CAT) has not yet been fully explored. This study thus aimed to develop an IRT-based tool for measuring depression in patients with cancer. We analyzed data from 393 patients with cancer from four tertiary centers in Japan who had not received psychiatric treatment. They answered 62 questions across five categories regarding their psychiatric status over the previous week. We selected 28 items that satisfied the assumptions of IRT, fitted a graded response model to these items, and performed CAT simulations. The CAT simulation used an average of 6.96 items and showed a Pearson’s correlation coefficient of 0.916 (95% confidence interval, 0.899–0.931) between the degree of depression estimated by simulation and that estimated using all 28 items. The measurement precision of CAT with only four items was superior to that of the estimation using the calibrated Patient Health Questionnaire-9. These results imply that this scale is useful and accurate for measuring depression in patients with cancer.

## Introduction

Depression frequently occurs in patients with cancer^1,2^. Even mild levels of depression reportedly decrease the quality-adjusted life-year score^3^. Furthermore, patients with cancer have a higher risk of suicide than the general population^4–6^. Several interventions, such as specialized palliative care, can reduce psychological symptoms^7,8^, thus, requiring accurate scales for measuring depression. Clinicians commonly evaluate the condition through self-administered tools, such as the Patient Health Questionnaire-9 (PHQ-9)^9,10^ and the Hospital Anxiety and Depression Scale (HADS)^11^, both of which are based on classical test theory. These have several disadvantages, including sample dependency, inability to replace or add items, and requirement for participants to answer all items regardless of severity. Scales developed based on item response theory (IRT) and computer adaptive testing (CAT) can address these limitations^12^. This is because IRT-based scales can reveal sample-independent subject traits, and CAT can optimize the way items are presented and reduce the associated burdens placed on patients. Several studies have applied CAT-based depression scales, which were developed in the Patient-Reported Outcomes Measurement Information System (PROMIS) project, to patients with cancer^13–16^. However, the usefulness of CAT-based scales for measuring depression in patients with cancer has not yet been fully explored. Thus, this study aimed to develop a CAT-based scale for measuring depression in patients with cancer.

## Methods

### Ethical approval

All participants provided written informed consent. The institutional review board of the National Cancer Center Hospital (approval number: 2010-202) and all the participating sites approved the study. This study was in accordance with the ethical standards of the 1964 Helsinki Declaration and its later amendments or comparable ethical standards.

### Study design and participants

This multicenter prospective study was conducted at four tertiary centers in Japan (National Cancer Center Hospital, National Cancer Center Hospital East, Okayama University Hospital, and the University of Tokyo Hospital) between May 2011 and December 2012. The study included patients who (a) were aged ≥ 20 years, (b) had been diagnosed with any type of cancer, (c) had Eastern Cooperative Oncology Group performance status ≤ 2, and (d) were selected for or were already receiving anti-cancer treatments. Patients who (a) had received psychiatric treatments within the previous two months, or (b) were considered extremely sick to participate by their physicians-in-charge were excluded from the study. We recruited participants upon admission.

### Data collection

We developed 62 items to measure depression. Table 1 shows examples of items translated from Japanese to English. Several psycho-oncologists independently drafted the items based on the diagnostic criteria and common symptoms of depression. Subsequently, they discussed and finalized these items. All items asked participants about their depressive mood over the preceding week, with each item answered on a 5-point scale (1 = none, 2 = rarely, 3 = sometimes, 4 = often, 5 = always).

**Table 1:** Examples of items translated from Japanese to English.

Items	Answer categories
I cannot focus on anything	1 = None; 2 = Rarely; 3 = Sometimes; 4 = Often; 5 = Always
I do not feel happy	1 = None; 2 = Rarely; 3 = Sometimes; 4 = Often; 5 = Always
I cannot enjoy my life	1 = None; 2 = Rarely; 3 = Sometimes; 4 = Often; 5 = Always

To confirm concurrent validity, participants completed the PHQ-9, which is widely used to measure depression and has been validated in patients with cancer^9,10^. The PHQ-9 total scores of 5–9, 10–14, 15–19, and 20–27 correspond to mild, moderate, moderately severe, and severe depression, respectively.

### Overview of statistical analyses

Based on the analytic methods used in the PROMIS project^17^ and several studies on CAT^18–20^, we conducted the following analyses: (1) descriptive statistics, (2) evaluation of the IRT assumptions, (3) fitting a graded response model (GRM) to the data, (4) evaluation of differential item functioning (DIF), (5) CAT simulations, and (6) calibration of the PHQ-9. All analyses were conducted using the open-source R software (version 4.1.1). Statistical significance was set at P < 0.05.

### Descriptive statistics

Cronbach’s alpha was used to measure internal consistency (analyzed using the R package “psych,” version 2.1.9). Items with unanswered categories were excluded because their parameters could not be estimated, and those with an item-remainder correlation < 0.3 were also excluded due to violation of internal consistency^21^.

### Evaluation of assumptions of the IRT model

We evaluated the assumptions of IRT including unidimensionality, local independence, and monotonicity^17^.

We tested unidimensionality by conducting principal component analysis (PCA), confirming that the proportion of variance of the first factor was ≥ 20% and the ratio of variance of the first factor to the second factor was ≥ 4^17,18^. We excluded items with a low contribution to the first factor to satisfy these criteria.

Subsequently, we tested local independence by conducting a one-factor confirmatory factor analysis, producing a residual correlation matrix (analyzed using the R package “lavaan,” version 0.6–9). From the pairs of items with residual correlations > 0.2^17,18^, we excluded the item with a lower contribution to the first factor of the PCA.

Finally, we tested monotonicity by developing a nonparametric IRT model (analyzed using the R package “mokken,” version 3.0.6), and excluded items with a scalability coefficient < 0.3^18^.

### Graded response model

We fitted a GRM to the remaining items (analyzed using the R package “mirt,” version 1.35.1) to estimate discrimination and difficulty parameters for each item and latent factor θ (i.e., degree of depression) for each patient using maximum a posteriori (MAP). Subsequently, we excluded items that contained categories without maximum probability at any θ. We also examined fit statistics (S-X^2^) for each item, excluding those with a poor fit, as determined at an alpha level of 0.01^17^.

### Evaluation of DIF

We evaluated DIF for age (≥ 65 or < 65) and sex (male or female) (analyzed using the “DIF” function in the R package “mirt,” version 1.35.1) and excluded items with an alpha level of 0.01^17,18^.

### CAT simulations

Following the item selection process, we recalculated Cronbach’s alpha, redeveloped a GRM, and recalculated discrimination and difficulty parameters for each item as well as θ for each patient (θ_true_).

We used the resulting items and θ_true_ to perform CAT simulations (analyzed using the R package “catIrt,” version 0.5–0)^19^. At the beginning of the simulations, the estimated latent factor (θ_est_) was set to zero, and the minimum number of items administrated was set to three. We conducted simulations using various combinations of latent factor estimators, item selection methods, and termination criteria.

Latent factor estimators were: (a) maximum likelihood estimation (MLE), (b) Bayesian modal estimation (BME), and (c) expected a priori estimation (EAP). Item selection methods were as follows: (a) unweighted Fisher information (UW-FI), and (b) pointwise Kullback–Leibler divergence (FP-KL). Termination criteria were: (a) standard error of measurement (SEM) threshold of 0.32 or (b) that of 0.50, while the simulations were also terminated upon reaching the maximum number of items.

We calculated Pearson’s correlation coefficients (PCCs) between θ_est_ and θ_true_ to measure the simulation accuracy, and PCCs between θ_est_ and the total score on the PHQ-9 to confirm concurrent validity.

### Calibration of PHQ-9 to the IRT model

To compare the measurement precision of the scale with that of the PHQ-9, we calibrated the PHQ-9 to the GRM model (analyzed using the “fixedCalib” function in the R package “mirt,” version 1.35.1), and performed an estimation using the calibrated items^20^. We plotted the Lowess curves of SEMs for the following: (a) CAT simulations with a fixed number of items and (b) estimation using the calibrated PHQ-9 items. Subsequently, we determined the minimum number of items required to surpass the measurement precision of the calibrated PHQ-9.

## Results

### Study participants

A total of 393 participants completed the questionnaires. The average score for all items was 1.44/5. The descriptive data are shown in Table 2. Among 289 patients who completed the PHQ-9, 77 (27%), 15 (5%), and 5 (2%) patients showed mild, moderate, and moderately severe to severe depression, respectively.

**Table 2:** Descriptive data of study participants.

Patients (N = 393)	
Age (years)	
Mean (SD) Median (range)	60.87 (13.54) 64 (20–84)
Sex, N (%)	
Male Female	265 (67) 128 (33)
The PHQ-9 total score	
Mean (SD) Median (range)	3.85 (3.74) 3 (0–23)
The PHQ-9 total score category, N (%)	
None–Minimal (0–4) Mild (5–9) Moderate (10–14) Moderately severe–Severe (15–27) Missing	192 (66*) 77 (27*) 15 (5*) 5 (2*) 104
Cancer type, N (%)	
Gastrointestinal Liver/binary tract/pancreas Lung Breast Genitourinary Hematological Other	3 (1) 83 (21) 56 (14) 6 (2) 100 (25) 51 (13) 94 (24)
Cancer stage, N (%)	
I II III IV Recurrent Other Undetermined	32 (8) 37 (9) 36 (9) 81 (21) 128 (33) 15 (4) 64 (16)
ECOG performance status, N (%)	
0 1 2 Missing	213 (54) 146 (37) 33 (8) 1 (0)

*The percentages are calculated excluding the missing data.

### Item selection and parameter estimation

**Figure 1:**
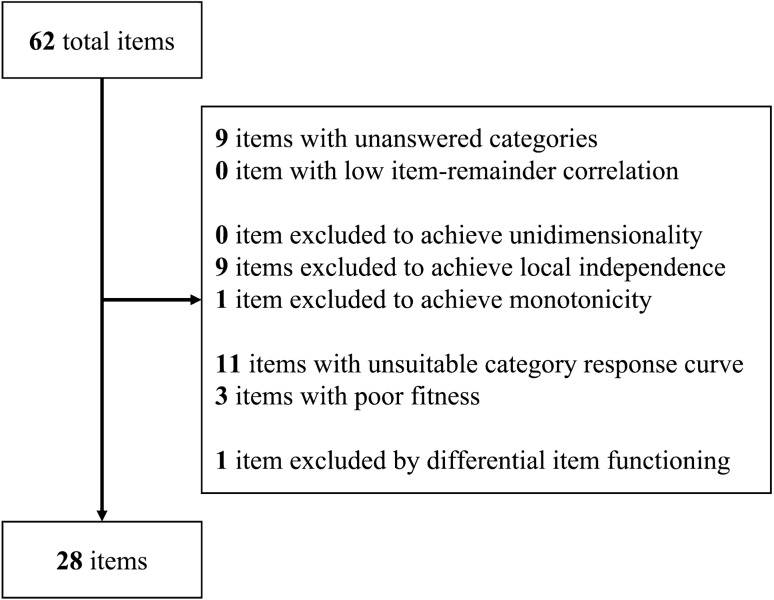
Flowchart of the item selection process. The flowchart shows the number of included and excluded items, as well as the reasons for exclusion.

Cronbach’s alpha for all 62 items was 0.97. As shown in Fig. 1, 28 of these items were included in the GRM and CAT simulations. Cronbach’s alpha was 0.95 after the item selection. Most unanswered categories comprised those with higher scores (4 or 5).

The examples of parameters in the GRM are shown in Table 3 (see Supplementary Table 1 for the parameters of all the items). Overall, the discrimination parameters ranged from 1.53 to 3.32. The first and last difficulty parameters ranged from 0.09 to 1.60 and 2.55 to 4.33, respectively. The item with the highest discrimination parameter was “I feel depressed and have difficulty in daily life.” The items with the lowest difficulty parameter were “I often feel helpless” and “I feel hopeless for the future.” The items with the highest difficulty parameter were “I need help with my depression” and “Others don't understand me”.

**Table 3:** Examples of estimated parameters.

Items	Discrimination	Difficulty			
	a	b_1_	b_2_	b_3_	b_4_
Highest discrimination (a)					
I feel depressed and have difficulty in daily life	3.32	0.93	1.84	2.32	3.33
Lowest b_1_ difficulty					
I often feel helpless	1.88	0.09	1.13	2.15	2.86
Lowest b_4_ difficulty					
I feel hopeless for the future	2.02	0.09	0.98	1.74	2.55
Highest first difficulty (b_1_)					
I need help with my depression	2.23	1.60	2.29	3.01	3.73
Highest last difficulty (b_4_)					
Others don't understand me	1.74	0.75	2.07	2.88	4.33

### CAT simulations

The results of the CAT simulations are presented in Table 4. When the termination criteria of the SEM threshold were set to 0.50, the most accurate simulation used the BME estimator and UW-FI item selection, achieving a PCC of 0.916 (95% confidence interval [CI], 0.899–0.931) using an average of 6.96 items. It also achieved a PCC with a total PHQ-9 score of 0.669 (95% CI, 0.600–0.728).

**Table 4:** Results of the computerized adaptive testing simulations.

Estimator	Item selection	Number of administrated items	PCC with θ_true_ (95% CI)
**SEM threshold θ of 0.32**			
MLE	UW-FI	13.98	0.892 (0.869–0.910)
MLE	FP-KL	13.99	0.892 (0.870–0.910)
BME	UW-FI	13.04	0.971 (0.965–0.976)
BME	FP-KL	13.05	0.973 (0.967–0.978)
EAP	UW-FI	13.70	0.975 (0.969–0.979)
EAP	FP-KL	13.72	0.975 (0.969–0.979)
**SEM threshold θ of 0.50**			
MLE	UW-FI	9.51	0.866 (0.839–0.889)
MLE	FP-KL	9.50	0.866 (0.839–0.889)
BME	UW-FI	6.96	0.916 (0.899–0.931)
BME	FP-KL	7.02	0.915 (0.897–0.930)
EAP	UW-FI	7.22	0.921 (0.905–0.935)
EAP	FP-KL	7.22	0.922 (0.905–0.935)

PCC, Pearson’s correlation coefficient; MLE, maximum likelihood estimation; BME, Bayesian modal estimation; EAP, expected a priori estimation; UW-FI, unweighted Fisher information; FP-KL, pointwise Kullback–Leibler divergence; CI, confidence interval.

**Figure 2:**
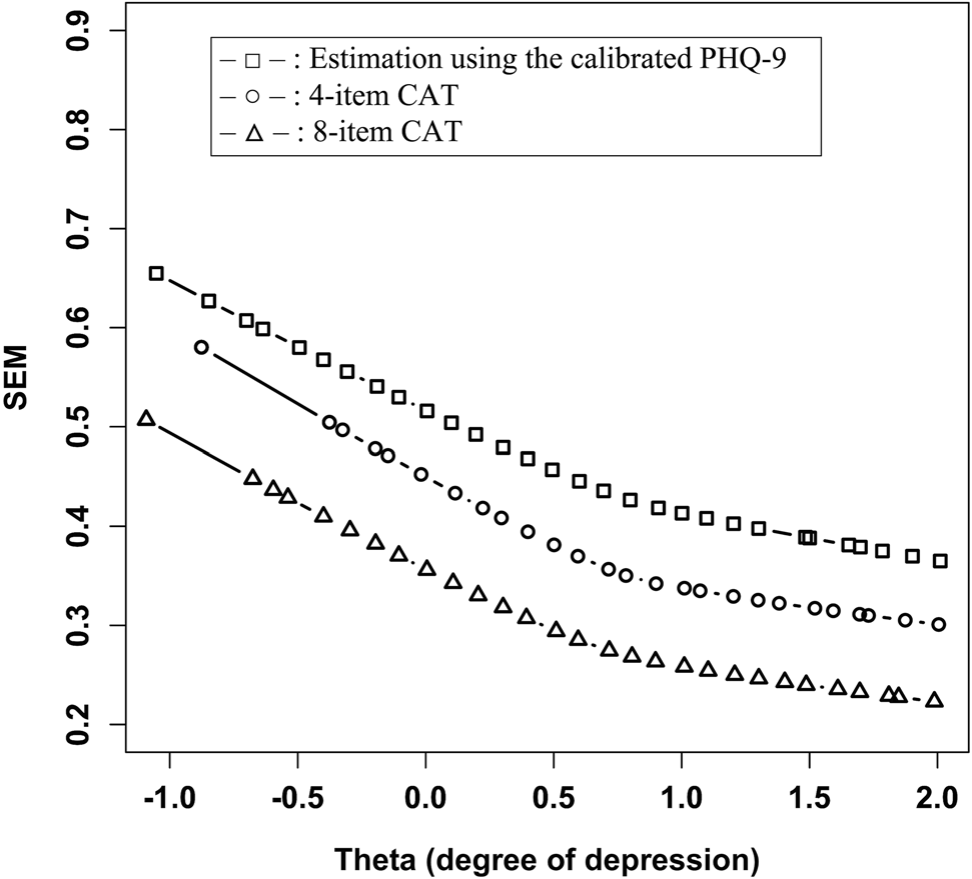
Lowess curves for the standard error of measurement (SEM) by θ (degree of depression). Each line indicates the Lowess curve for SEMs in each estimation (□, estimation using the calibrated PHQ-9; ○, 4-item CAT; △, 8-item CAT). The measurement precision of CAT using only four items surpassed that of the estimation using the calibrated PHQ-9.

The Lowess curves for the SEMs of the CAT simulations are shown in Fig. 2. CAT using only four items had smaller SEMs at any θ_est_ than the estimation using the calibrated PHQ-9. The estimated parameters of PHQ-9 are listed in Supplementary Table 2.

## Discussion

We developed a new scale for measuring depression in patients with cancer based on an IRT model and CAT simulations. The CAT simulations showed that a small number of items could accurately measure the degree of depression. The scale also showed a significant correlation with the PHQ-9 total score and achieved a smaller SEM than the calibrated PHQ-9 using only four items.

More than half of the items were excluded through the item selection process. The main reasons for this included unanswered categories, violations of local independence, and unsuitable category response curves. The existence of unanswered categories, which mainly comprised those with higher scores, may have resulted from the exclusion of patients undergoing psychiatric treatment. The existence of local dependence in many items might suggest duplication or redundancy in our item development. The unsuitable category response curves may have resulted from sample size insufficiency because the GRM requires more than 500 samples to estimate the parameters accurately^22^. The remaining 28 items exhibited a Cronbach’s alpha of 0.95, suggesting substantial internal consistency^23^.

The exclusion of more than half of the items may also be attributable to the item selection using the unidimensional model. Instead, the bifactor model applied for larger item banks would be beneficial for developing CAT with more items. Gibbons et al. showed that such an analysis could result in the development of a CAT measuring depression/anxiety with hundreds of items^24,25^. Such an analysis would also be necessary for our aim to develop CAT measuring depression in patients with cancer.

The parameters of several items may explain the characteristics of depression in patients with cancer. The discrimination parameter corresponds to the slope of the GRM and indicates the ability to discriminate subjects’ traits. The highest discriminative item was about the influence on daily life. Such an influence appears highly informative for assessing depression in patients with cancer. A previous study, which assessed depression in patients with cancer using IRT, showed that social withdrawal or decreased talkativeness is highly discriminative^26^, which is similar to the result of the present study. However, other IRT-based studies on depression in patients with cancer did not include items that assessed the influence on daily lives^27–29^. Thus, the importance of this item needs to be further examined.

The difficulty parameter indicates the traits of participants at which the probability of choosing either of the two adjacent categories is equal. Thus, items with high difficulty parameters were selected by participants with high severity, whereas items with low difficulty parameters were selected even by participants with low severity. The items about helplessness and hopelessness showed low difficulty parameters, suggesting that patients with cancer easily experience these symptoms. In contrast, the items about support and understanding from others showed high difficulty parameters, suggesting that these symptoms would be observed in highly depressive patients with cancer. These items were not included in previous IRT-based studies on depression in patients with cancer^26–29^. In addition, the item selection process may have excluded items with higher difficulty or those with less difficulty. Thus, further studies are required to determine the importance of these items.

The CAT simulations achieved high measurement accuracy using a small number of items, exhibiting strength in shortening the health measurement scales. The significant correlation with the PHQ-9 score implies the ability of the scale to measure depression. Moreover, the CAT simulations showed a higher measurement accuracy than the estimation using the calibrated PHQ-9. Thus, the scale can be employed in clinical settings to efficiently evaluate depression in patients with cancer. The CAT developed in this study could be made available online, as in the PROMIS project, which would allow efficient assessment of depression in patients with cancer to be applied in clinical settings, such as palliative care and psycho-oncology.

This study has several limitations that need to be addressed. First, we excluded patients who were undergoing psychiatric treatments, which may have affected item selection and limited the situations of the CAT’s usage. Second, the sample size was insufficient. GRM reportedly requires more than 500 samples to estimate parameters of 25 items appropriately^22^. However, we recruited only 393 participants to estimate the parameters of 62 items. Third, we could not perform a DIF analysis for the history of depression because only few participants had it, which is likely due to the exclusion of participants under psychiatric treatments. Fourth, the final item set is limited, and only a small number of them are available for adaptive administration at each level of depression severity. Fifth, the moderate correlation between the CAT and the PHQ-9, with a correlation coefficient of 0.67, may imply inadequate measurement of depression by the scale. This result might also suggest that the items did not cover all subdomains of depression, such as somatic symptoms. Further investigation is necessary to examine the correlation between the CAT and the HADS. Finally, we did not confirm that the scale could accurately classify whether a patient has a major depressive disease or not. Several studies examined the diagnostic performance of the developed CAT for patients diagnosed through gold standard measures, such as structured interviews^24,25^. Such examination for diagnostic ability is also required for the newly developed CAT in the present study.

In conclusion, this study developed a scale for measuring depression in patients with cancer based on IRT and CAT, providing a useful and improved way for clinicians to evaluate depression in patients with cancer.

## Supplementary Information

Supplementary Information.

## Data Availability

The datasets analyzed during the current study are not publicly available because the approval of data sharing has not been obtained from the institutional review board but are available from the corresponding authors on reasonable request.
