# Supplementary material for: Development of computer adaptive testing for measuring depression in patients with cancer
Source: Sci Rep. 2022 May 17;12:8247. doi: 10.1038/s41598-022-12318-x (PMC9114408; doi:10.1038/s41598-022-12318-x)
Supplement: Supplementary file 1 — Supplementary Information. [file 41598_2022_12318_MOESM1_ESM.docx]

**Supplementary Table 1. Estimated parameters in GRM of remaining items (translated from Japanese to English)**

|  | Discrimination | Difficulty | | | |
| --- | --- | --- | --- | --- | --- |
| Items | a | b_1_ | b_2_ | b_3_ | b_4_ |
| I cannot get rid of depression regardless of help from family or friends. | 1.84 | 0.74 | 1.60 | 2.43 | 3.39 |
| I cannot concentrate. | 1.78 | 0.13 | 1.08 | 2.13 | 3.29 |
| I feel down. | 2.43 | 0.16 | 1.22 | 2.06 | 3.14 |
| I feel hopeless for the future. | 2.02 | 0.09 | 0.98 | 1.74 | 2.55 |
| I cannot feel happy. | 2.26 | 0.52 | 1.42 | 2.14 | 2.77 |
| I talk less than usual. | 1.89 | 0.47 | 1.25 | 2.29 | 3.50 |
| I feel lonely. | 2.21 | 0.23 | 1.18 | 2.07 | 2.84 |
| I cannot enjoy my daily life. | 2.13 | 0.35 | 1.21 | 2.07 | 2.84 |
| I feel sad. | 1.87 | 0.39 | 1.54 | 2.52 | 3.58 |
| I feel depressed. | 2.72 | 0.24 | 1.22 | 2.14 | 2.79 |
| I cannot enjoy things that I used to. | 1.97 | 0.40 | 1.19 | 1.95 | 2.71 |
| I'm not interested in my own appearance. | 1.71 | 0.83 | 1.55 | 2.19 | 2.93 |
| I cannot enjoy books, TV, or radio. | 1.87 | 0.81 | 1.59 | 2.47 | 3.36 |
| I have decreased interest in my surroundings. | 2.23 | 0.46 | 1.28 | 2.17 | 3.10 |
| I often feel helpless. | 1.88 | 0.09 | 1.13 | 2.15 | 2.86 |
| I feel hopeless. | 2.25 | 0.77 | 1.55 | 2.46 | 3.15 |
| I feel guilty. | 1.53 | 1.16 | 2.32 | 3.26 | 4.18 |
| I feel miserable. | 2.77 | 0.84 | 1.60 | 2.41 | 3.06 |
| I'm unwilling to interact with others. | 1.90 | 0.68 | 1.63 | 2.42 | 3.16 |
| I have mood swings. | 2.05 | 0.09 | 1.11 | 2.09 | 3.56 |
| Others don't understand me. | 1.74 | 0.75 | 2.07 | 2.88 | 4.33 |
| I'm disappointed in myself. | 2.41 | 1.03 | 1.75 | 2.66 | 3.71 |
| I feel my life is empty. | 2.39 | 0.97 | 1.88 | 2.60 | 3.15 |
| I cannot think straight. | 1.90 | 0.51 | 1.88 | 2.97 | 3.71 |
| I'm mentally exhausted. | 2.32 | 0.79 | 1.84 | 2.62 | 3.79 |
| I need help with my depression. | 2.23 | 1.60 | 2.29 | 3.01 | 3.73 |
| I often sigh. | 1.68 | 0.51 | 1.89 | 2.93 | 3.76 |
| I feel depressed and have difficulty in daily life. | 3.32 | 0.93 | 1.84 | 2.32 | 3.33 |

**Supplementary Table 2. Estimated parameters of the calibrated PHQ-9**

|  | Discrimination | Difficulty | | |
| --- | --- | --- | --- | --- |
| Items | a | b_1_ | b_2_ | b_3_ |
| 1 | 2.06 | 0.58 | 2.07 | 3.01 |
| 2 | 1.98 | 0.54 | 2.12 | 3.05 |
| 3 | 0.56 | -1.05 | 1.89 | 4.14 |
| 4 | 1.55 | 0.01 | 1.82 | 2.87 |
| 5 | 1.06 | 0.45 | 2.24 | 3.88 |
| 6 | 1.46 | 0.80 | 1.99 | 3.01 |
| 7 | 1.36 | 1.15 | 2.77 | 3.33 |
| 8 | 1.14 | 1.99 | 4.27 | 4.90 |
| 9 | 1.90 | 2.34 | 3.57 | N.A. |
